# Oncolytic virus: A catalyst for the treatment of gastric cancer

**DOI:** 10.3389/fonc.2022.1017692

**Published:** 2022-11-24

**Authors:** Junqing Wang, Linyong Du, Xiangjian Chen

**Affiliations:** ^1^ School of the 1st Clinical Medical Sciences, Wenzhou Medical University, Wenzhou, Zhejiang, China; ^2^ Key Laboratory of Laboratory Medicine, Ministry of Education of China, School of Laboratory Medicine and Life Science, Wenzhou Medical University, Wenzhou, Zhejiang, China

**Keywords:** gastric cancer, oncolytic virus, adenovirus, herpes simplex virus type 1, tumor microenvironment, combination therapy

## Abstract

Gastric cancer (GC) is a leading contributor to global cancer incidence and mortality. According to the GLOBOCAN 2020 estimates of incidence and mortality for 36 cancers in 185 countries produced by the International Agency for Research on Cancer (IARC), GC ranks fifth and fourth, respectively, and seriously threatens the survival and health of people all over the world. Therefore, how to effectively treat GC has become an urgent problem for medical personnel and scientific workers at this stage. Due to the unobvious early symptoms and the influence of some adverse factors such as tumor heterogeneity and low immunogenicity, patients with advanced gastric cancer (AGC) cannot benefit significantly from treatments such as radical surgical resection, radiotherapy, chemotherapy, and targeted therapy. As an emerging cancer immunotherapy, oncolytic virotherapies (OVTs) can not only selectively lyse cancer cells, but also induce a systemic antitumor immune response. This unique ability to turn unresponsive ‘cold’ tumors into responsive ‘hot’ tumors gives them great potential in GC therapy. This review integrates most experimental studies and clinical trials of various oncolytic viruses (OVs) in the diagnosis and treatment of GC. It also exhaustively introduces the concrete mechanism of invading GC cells and the viral genome composition of adenovirus and herpes simplex virus type 1 (HSV-1). At the end of the article, some prospects are put forward to determine the developmental directions of OVTs for GC in the future.

## 1 Introduction

Gastric cancer (GC) is a common malignant tumor originating from gastric mucosa epithelial cells, and its onset is relatively insidious. In the early stage, there is generally no apparent symptom of discomfort or only indigestion-like clinical manifestations such as inappetence, gastroesophageal reflux, belching, and stomachache. However, as the disease progresses, hemorrhage, perforation, obstruction, cachexia, and other symptoms of advanced gastric cancer (AGC) gradually appear. Unfortunately, patients are already in the terminal stage at this time, and the cancer cells have already invaded the surrounding organs or metastasized far away, which leads to the loss of the curative chance for most patients (1). According to the GLOBOCAN 2020 estimates of incidence and mortality for 36 cancers in 185 countries produced by the International Agency for Research on Cancer (IARC), an estimated 19.3 million new cancer cases and almost 10.0 million cancer deaths occurred worldwide in 2020, and GC ranked fifth (5.6%) and fourth (7.7%), respectively (2). Therefore, accurately diagnosing early GC and effectively treating AGC patients who have lost the chance of radical surgical resection are two serious health problems worldwide.

Currently, therapeutic strategies for GC mainly incorporate surgical resection, radiotherapy, chemotherapy, targeted therapy, and immunotherapy. Among them, radical surgical resection is still the preferred and only method considered to cure GC. Endoscopic submucosal dissection (ESD) is recommended for early GC which is limited to the mucosa and without lymph node metastasis, and gastrectomy with D2 lymphadenectomy is recommended for AGC (3). Radiotherapy, chemotherapy, targeted therapy, and immunotherapy are basically intended to provide another choice for patients with post- or non-operative conditions. Chemotherapy, as the most common and indispensable adjuvant therapy, is updated constantly with advances in research. Recently, a randomized, open-label, phase 3 trial (NCT02322593) in 62 centers across Japan and South Korea showed that TAS-118 (S-1 (an oral anticancer agent comprising the 5-fluorouracil prodrug tegafur and targeted modulators, gimeracil and oteracil) plus leucovorin) plus oxaliplatin is more effective than S-1 plus cisplatin, and could be considered a new first-line treatment option for AGC in Asian patients (the median overall survival (OS) was 16.0 months (95% CI 13.8–18.3) in the TAS-118 plus oxaliplatin group and 15.1 months (95% CI 13.6–16.4) in the S-1 plus cisplatin group (hazard ratio 0.83, 95% CI 0.69–0.99; p=0.039)) (4). Furthermore, targeted therapy usually focuses on the human epidermal growth factor receptor 2 (HER2), because of its frequent amplification (5). In an open-label, randomized, phase 2 trial (NCT03329690), therapy with trastuzumab led to significant improvements in response and OS, compared with standard chemotherapy, among patients with HER2-positive GC (median, 12.5 vs. 8.4 months; hazard ratio for death, 0.59; 95% confidence interval, 0.39 to 0.88; P=0.01) (6). Although chemotherapy and targeted therapy have obtained satisfactory efficacy, resistance and fatal side effects gradually develop with the extension of exposure time. Thus, a new strategy is urgently needed to fill this gap.

With the exploration of the biological behavior and internal molecular mechanism of cancers, immunotherapy has become a novel, popular and promising treatment that can restore the ability of the immune system to respond to neoplasms, limiting their growth and killing malignant cells, ultimately achieving remission or even complete elimination (7). Existing cancer immunotherapies mainly include tumor vaccines (such as cervical cancer vaccine), immune checkpoint inhibitors (ICIs) (such as anti-PD-L1 antibody), adoptive immunotherapies (such as CAR T-cell) and OVTs (such as adenovirus) (8). OVTs are a safe and mature immunotherapy method because of their representative capacity to promote the infiltration of immune effector cells into the tumor microenvironment (TME) and have been favored by numerous researchers (9).

OVs, as their name implies, can specifically invade tumor cells and eventually lyse them. The first definitive record of OV was in 1904, when a 42-year-old leukemia patient was infected with influenza. Doctors were surprised to find a dramatic decrease in malignant cells in his blood. Recently, on January 2, 2021, Sarah Challenor also reported a 61-year-old patient with stage III Epstein–Barr virus (EBV)-positive classical Hodgkin lymphoma who was diagnosed with COVID-19, and four months later, the palpable lymphadenopathy had reduced and a fluorine-18 fluorodeoxyglucose positron emission tomography/CT (^18^FDG-PET/CT) scan revealed widespread resolution of the lymphadenopathy and reduced metabolic uptake throughout (10). These miraculous phenomena about viruses “curing” cancers suggest that OVTs may be a promising cancer treatment. More notably, in 2015, the US Food and Drug Administration (FDA) approved the first OV (T-Vec, a HSV-1-based OV generated by deleting ICP47 gene and replacing ICP34.5 with GM-CSF gene) for the clinical treatment of malignant tumors by virtue of a randomized, open-label, phase 3 trial (NCT00769704); it can achieve a higher durable response rate (DRR) (26.4%; 95% CI, 21.4% to 31.5% vs. 5.7%; 95% CI, 1.9% to 9.5%) and longer OS (23.3 months; 95% CI, 19.5 to 29.6 months vs. 18.9 months; 95% CI, 16.0 to 23.7 months) in patients with unresectable stage IIIB to IV melanoma than subcutaneous injection of granulocyte macrophage colony-stimulating factor (GM-CSF) alone (11). In Japan, another single-arm, phase II clinical trial (UMIN000015995) was completed to test the efficacy of G47Δ (a HSV-1-based OV generated by deleting ICP47 and ICP34.5 genes and replacing ICP6 gene with lacZ coding sequence) administered stereotactically in patients with residual or recurrent glioblastoma, and the results indicated that the 1-year- survival rate of 13 patients reached an astonishing 92.3% (12). Moreover, in a phase I/II, single-arm study (UMIN000002661) assessing the safety of G47Δ, the results revealed that G47Δ was safe for treating recurrent or progressive glioblastoma and warranted further clinical development (13). Based on these, G47Δ has received conditional approval from Japan’s Ministry of Health, Labor and Welfare (MHLW) as an OVT for patients with malignant glioma. Furthermore, studies proving the ability of OVTs to rapidly eliminate cancer cells have led to approval of H101 in China and Rigvir in Latvia.

An increasing number of OVTs are being clinically approved, and trials of OVs for other malignancies have sprung up continually, including GC. As a malignant tumor with a poor prognosis, strong heterogeneity, and low immunogenicity, GC therapy may acquire a certain degree of breakthrough with the help of OV’s exceptional function. Moreover, peritoneal metastasis is the most frequent form of distant metastasis and recurrence in GC, and the prognosis is extremely poor due to the resistance of systemic chemotherapy. OVTs for GC patients with peritoneal metastases *via* intraperitoneal injection not only act on the vast majority of metastases, but also activate the inherent immune cells in the abdominal cavity and recruit immune cells in the blood to exert corresponding antitumor functions. However, there is no systematic summary of OVTs for GC to date. In this review, we integrate most of the experimental studies and clinical trials of various OVs tested for the diagnosis and therapy of GC and meticulously discuss the mechanism of infection and the viral genome composition of adenovirus and HSV-1. Finally, we also put forward some prospects about the developmental directions of OVTs for GC in the future.

## 2 Mechanism of OVTs

According to existing experimental results on OVTs for cancers, a nonoptimal delivery route is one of the dominant reasons for treatment failure. Intravenous administration is a simple, common, and effective route for other cancer treatments, but when OVs are injected into the bloodstream, viral defense barriers such as the complement system, immunoglobulin and coagulation cascade will promptly inactivate the virus particles (14). Virions marked by natural immunoglobulin M (IgM) antibodies and coagulation factor X are captured and cleared by macrophages rapidly and efficiently in the liver, spleen, and lung, which probably trigger toxic reactions in the corresponding organs (15). Furthermore, during the process of rapid proliferation of cancer cells, a suitable environment for their survival called the tumor microenvironment (TME) is gradually established. Numerous mesenchymal cells such as myeloid-derived suppressor cells (MDSCs), cancer-associated fibroblasts (CAFs), mesenchymal stem cells (MSCs), and capillary endothelial cells, which are abundant in the TME, constitute a physical barrier in conjunction with the extracellular matrix (ECM). Even if OVs successfully escape the abovementioned clearance process and enter the TME, they will ultimately be captured or rejected by these “trap” cells, resulting in a further decrease in oncolytic effectiveness (16). Thus, intratumoral injection is still the optimal route of administration for solid tumors, including GC (17).

**Figure 1:**
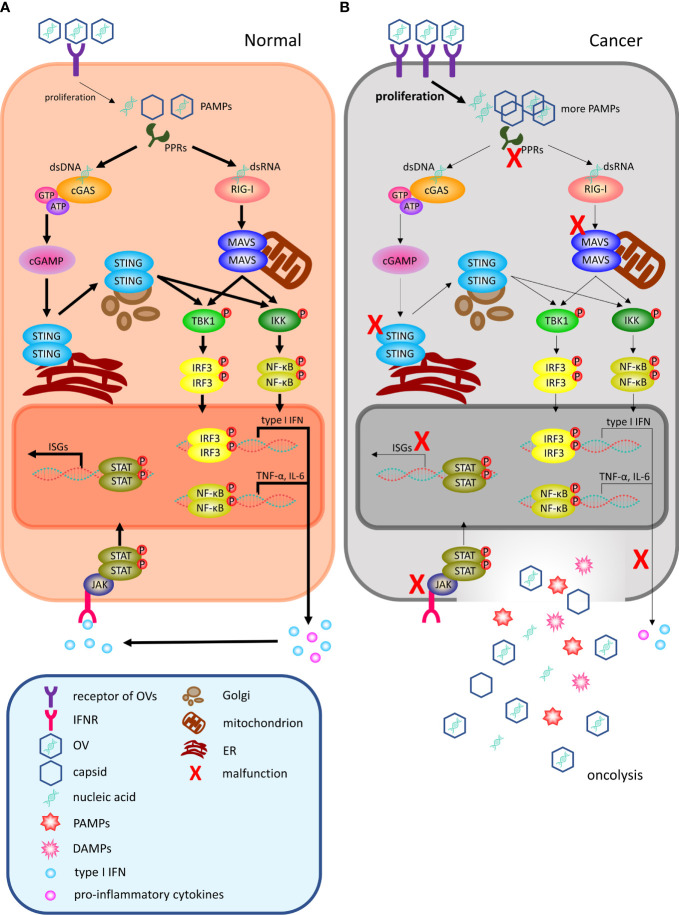
OVs can selectively infect and lyse cancer cells locally. **(A)** Following viral infection, most normal cells activate antiviral pathways against viral infections. The antiviral machinery can be triggered by viral PAMPs that activate PRRs (such as cGAS for DNA viruses and RIG-I for RNA viruses). Once PAMPs are detected, a signaling cascade through the adaptor molecule STING or MAVS phosphorylating IRF3 and NF-κB to dimerize and translocate to the nucleus to regulate the programmed transcription of type I IFN and proinflammatory cytokines. Among them, proinflammatory cytokines recruit immune cells to infiltrate the TME, and local IFN production can promote antiviral activity through IFNR. Upon type I IFN binding to receptors, the activated JAK-STAT signaling pathway leads to the rapid transcription of abundant ISGs to inhibit various stages of the viral lifecycle from invasion to release and can even target infected cells for apoptosis or necrosis. **(B)** In malignant cells, this process is disrupted. Cancer cells may increase the number of viral receptors or downregulate key signaling components within the innate antiviral signaling pathway, including PPRs, STING, MAVS, type I IFN and ISGs, thereby limiting their proapoptotic and cell cycle regulatory effects. Therefore, OVs can easily reach the critical value of viral load for oncolysis. OV, oncolytic virus; PAMPs, pathogen-associated molecular patterns; DAMPs, damage-associated molecular patterns; PRRs, pattern-recognition receptors; dsDNA, double-stranded linear DNA; dsRNA, double-stranded linear RNA; cGAS, cyclic GMP–AMP synthase; ATP, adenosine triphosphate; GTP, guanosine triphosphate; cGAMP, cyclic GMP–AMP; ER, endoplasmic reticulum; TBK1, TANK-­binding kinase 1; IKK, IκB kinase; IRF3, interferon regulatory factor 3; NF-κB, nuclear factor-κB; RIG-I, retinoic acid-inducible gene I; TNF-α, tumor necrosis factor-α; IL-6, interleukin-6; ISGs, interferon-stimulated genes; IFN, interferon; IFNR, interferon receptor; JAK, Janus kinase; STAT, signal transducer and activator of transcription; TME, tumor microenvironment.

**Figure 2:**
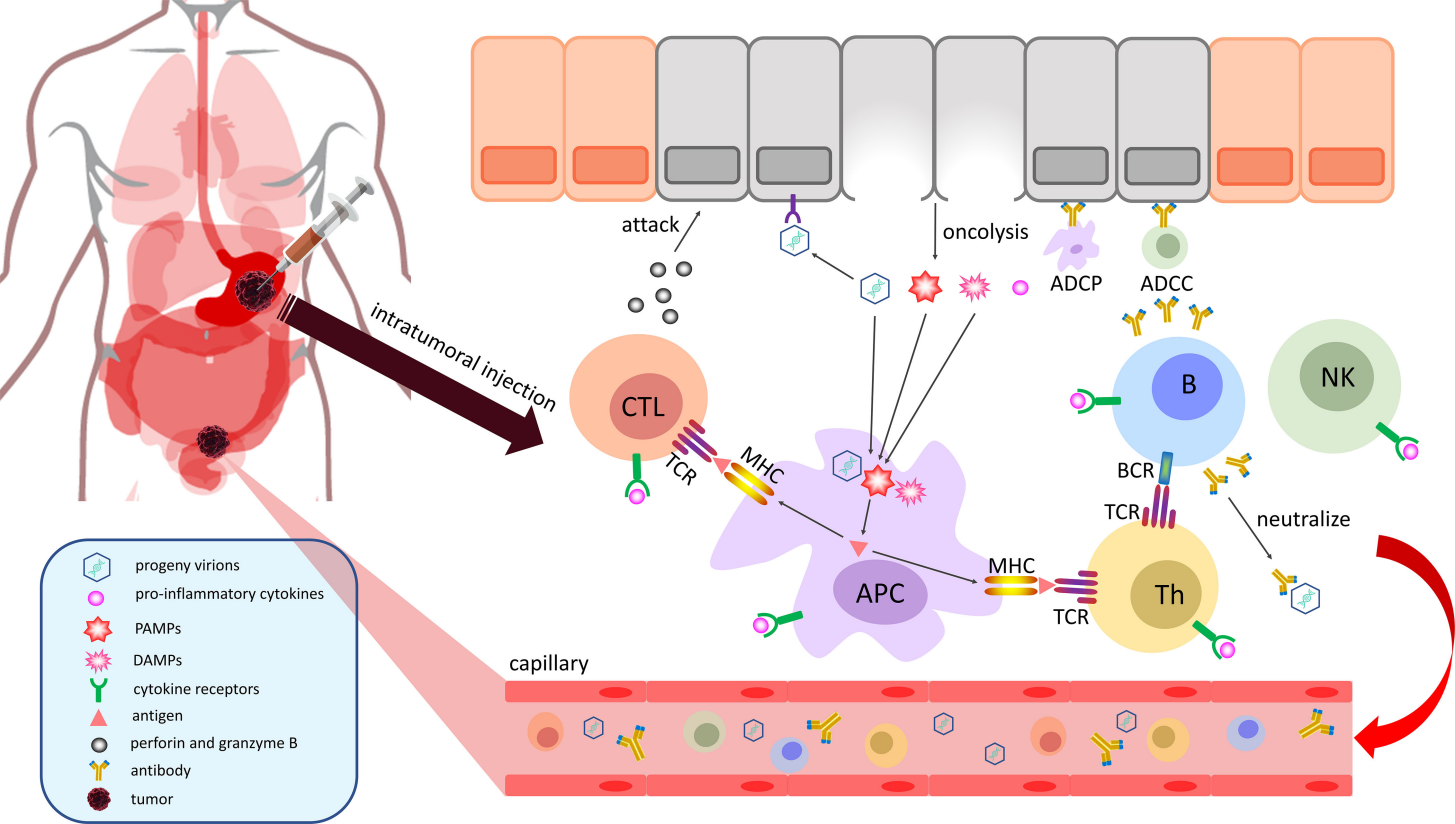
OVs can stimulate a systemic adaptive antitumor immune response. Cancer cells are lysed by mature OVs to release viral progeny, TAAs, PAMPs and DAMPs into the TME. Among them, progeny virions will ceaselessly infect the surrounding cancer cells to establish a cascade amplification reaction to eliminate malignancy. Infiltrative APCs can swallow and process TAAs, PAMPs, and DAMPs to present neoantigens by MHC molecules for the activation of immune cells. The sensitized CTLs attack the identified cancer cells by releasing perforin and granzyme B, and the activated Th cells can stimulate B cells to promote their activation and secrete neutralizing antibodies, which can mark malignant cells for ADCC by NK cells or ADCP by macrophages. Finally, immune effector cells, immune effector molecules and progeny virions will travel through the body with the blood to initiate a systemic adaptive antitumor immune response. APC, antigen-presenting cell; NK, natural killer cell; MHC, major histocompatibility complex molecule; CTL, cytotoxic T lymphocyte; Th, T helper cell; ADCC, antibody-dependent cellular cytotoxicity; ADCP, antibody-dependent cellular phagocytosis; PAMPs, pathogen-associated molecular patterns; DAMPs, damage-associated molecular patterns; TCR, T-cell receptor; BCR, B-cell receptor; TAA, tumor-associated antigen.

When OVs invade the interior of tumors, they mainly exert their antitumor function by selectively infecting and lysing the malignant cells locally (**Figure 1**) and stimulating the systemic adaptive antitumor immune response (**Figure 2**) (18):

### 2.1 Selectively infect and lyse the malignant cells locally

First, OVs infect host cells by recognizing and combining with relevant receptors or other special routes. Subsequently, they replicate and amplify by using nutrients from the host, similar to other viruses (18). After a certain period of proliferation, a small amount of new progeny virions, viral nucleic acids, and capsid protein accumulate inside the cell, which are known as pathogen-associated molecular patterns (PAMPs), and these products are identified by pattern-recognition receptors (PRRs, including Toll-like receptor (TLR), NOD-like receptor (NLR), and RIG-I-like receptor (RLR)) to initiate innate antiviral immune responses (19). In this review, we mainly highlight the two most acknowledged antiviral signal transduction pathways.

For a DNA virus, its gene fragments can activate the DNA sensor cyclic GMP–AMP synthase (cGAS) through direct binding, which triggers conformational changes that induce enzymatic activity (**Figure 1A**). Activated cGAS converts GTP and ATP into cyclic GMP–AMP (cGAMP), which is a unique endogenous second messenger. Then, the cGAMP product binds to STING, an endoplasmic reticulum (ER) -localized adaptor, and undergoes a conformational change to form dimers. Following the translocation of STING dimers to the Golgi apparatus, they interact with TANK­binding kinase 1 (TBK1) and IκB kinase (IKK), which phosphorylate interferon regulatory factor 3 (IRF3) and nuclear factor-κB (NF-κB), respectively. Activated IRF3 and NF-κB dimerize and translocate to the nucleus to regulate the transcription of type I interferon (IFN) and proinflammatory cytokines (20). In addition, other viral DNA sensors, such as IFI16, DAI and DDX41, can transmit antiviral immune signals through STING (21).

Retinoic acid-inducible gene I (RIG-I) is one of the primary originators of initiating infection signals of RNA virus, the other antiviral signal transduction pathway, along with melanoma differentiation association gene 5 (MDA5) and laboratory of genetics and physiology 2 (LGP2) (22). In the absence of ligand, RIG-I exists in an autorepressed conformation wherein its helicase domain and repressor domain (RD) associate with its caspase activation and recruitment domains (CARDs), which precludes it from participating in signaling. It is only when RIG-I engages the appropriate PAMP RNA through the helicase and RD that the CARDs are released from autorepression to associate with the adaptor molecule mitochondrial antiviral-signaling protein (MAVS) on the membrane surface of mitochondria. MAVS assembles into aggregates that allow the ensuing signaling cascade to induce the phosphorylation and nuclear translocation of the key innate immune transcription factors IRF3 and NF-κB to drive the expression of downstream genes (23).

In summary, both DNA and RNA viruses can induce the production of type I IFN, which is released into the microenvironment surrounding infected cells. Upon type I IFN binding to receptors, a signal is transmitted by activating the Janus kinase signal transducer and activator of transcription (JAK-STAT) pathway in the cells, leading to the rapid transcription of abundant IFN-stimulated genes (ISGs), such as myxovirus resistance (Mx), viperin, and double-stranded RNA-dependent protein kinase (PKR), and they can inhibit various stages of the viral lifecycle from invasion to release (24). When normal cells are infected with OVs, the intact innate antiviral immune system responds quickly and eliminates internal immature virions, sometimes even inducing apoptosis of seriously infected cells to protect the other cells.

However, during the process of becoming cancerous, some aberrant changes enable OVs to survive and proliferate extensively in host cells (**Figure 1B**); for example, the number of viral receptors on the membrane surface increases dramatically. Adenovirus, as a familiar OV, mainly engages its receptor coxsackievirus adenovirus receptor (CAR) and coreceptor integrins to complete the invasion process (25), and the expression of CAR is significantly elevated in GC, lung cancer and female reproductive tumors (26, 27). Similarly, the herpes virus receptor, herpesvirus-entry mediator (HVEM), is also markedly increased in malignant melanoma, colorectal cancer, GC, and glioblastoma (28). The amplification of these receptors will facilitate an increasing number of virions entering the host cells and increase the basic level of OVs to obviously accelerate the multiplication rate. Moreover, PPRs act as viral sensors and cannot efficaciously activate their downstream signal of viral defense if their expression is reduced or their function is destroyed. In hepatocellular carcinoma (HCC), TLRs are downregulated to protect cancer cells from the apoptosis they trigger, likely linked to the occurrence and poor prognosis of HCC (29). Similarly, recent studies have demonstrated that NLRs function as intrinsic tumor suppressors in intestinal epithelial cells (IECs), by regulating their responses to proliferative signals following intestinal injury, but they are frequently deleted in colorectal cancer (30). Adaptors STING and MAVS are indispensable intermediate transducers of antiviral signals, and the existing data imply that the STING signaling pathway may be recurrently suppressed by multifarious mechanisms in a considerable variety of malignant diseases and may be required for cellular transformation (31). To the best of our knowledge, cancers mainly acquire energy through glycolysis due to their rapid growth rate. Lactate serves as a key metabolite responsible for glycolysis-mediated RLR signaling inhibition by directly binding to the MAVS transmembrane domain and preventing MAVS aggregation, building a barrier to impede type I IFN production upon RLR activation (32). Hypoxia is also a common phenomenon in solid tumors and is strongly linked to hallmarks of cancers. This will lead to an overall downregulation of the type I IFN pathway to block the transcription of ISGs, due to repressed transcription and lower chromatin accessibility in a hypoxia-inducible factor 1/2α-independent manner (33). Cancer cells have a significantly weakened defense capability against OVs through the abovementioned various adaptive changes, which allow OVs to replicate and assemble, safely and quickly. Coupled with the silencing or mutation of genes that mediate apoptosis signals, infected tumor cells will not die immediately under normal circumstances (34, 35). Therefore, OVs can easily reach the critical value of viral load in tumor cells, eventually lysing them and releasing progeny viruses into the TME (34). Overall, OVs can take advantage of the differences in affinity and tolerance between normal and malignant cells for selectively infecting and lysing cancer cells.

### 2.2 Stimulate the systemic adaptive antitumor immune response

The immunogenicity of oncolysis caused by overloaded OVs significantly exceeds the process of apoptosis, which can stimulate a systemic antitumor immune response to a certain extent (**Figure 2**) (36). Findings from a phase II, multicenter, open-label study (NCT02366195) of patients with stage IIIB–IVM1c melanoma indicated that T-Vec had a significant therapeutic effect at the injection site of the tumor and it upregulated immune-cell populations in noninjected lesions, such as CD8^+^ and CD4^+^ T cells (37). When cancer cells cleaved by OVs undergo immunogenic cell death (ICD), a large number of progeny virions, tumor-associated antigens (TAAs), PAMPs and damage-associated molecular patterns (DAMPs) are released into the TME and blood circulation (18). Therefore, progeny virions will ceaselessly infect the surrounding cancer cells to establish a cascade amplification reaction, ultimately achieving the purpose of eliminating the malignancy. Proinflammatory cytokines produced by activated NF-κB can recruit antigen-presenting cells (APCs), B cells, T cells and natural killer (NK) cells and stimulate the activation of their relevant signaling pathways to perform antitumor functions (38). APCs, as specialized antigen-presenting cells, can take up and process the TAAs, PAMPs, and DAMPs produced by oncolysis and present these peptide antigens to T cells by major histocompatibility complex (MHC) molecules, ultimately activating CD4^+^ T cells and CD8^+^ T cells with the participation of costimulating molecules on the membrane surface (39). The sensitized cytotoxic T lymphocytes (CTLs) attack identified cancer cells by releasing perforin and granzyme B (18), and activated T helper (Th) cells offer costimulatory signals to B cells, thereby promoting their activation, causing them to secrete neutralizing antibodies, which can mark malignant cells for antibody-dependent cellular cytotoxicity (ADCC) by NK cells or antibody-dependent cellular phagocytosis (ADCP) by macrophages (40). In the end, immune effector cells, immune effector molecules and progeny virions generated in the TME will travel through the body with the blood and initiate a systemic adaptive antitumor immune response.

### 2.3 Two major therapeutic strategies of OVTs

The existing therapeutic strategies of OVTs are mainly divided into two categories, one is an oncolytic tool relies on its oncolysis, another is an exogenous gene delivery system relies on its selectivity for various cancers. As mentioned above, natural OVs can preferentially replicate and assemble in human cancer cells and inhibit tumor growth without specific deletion or modification to the genome. Multiple preclinical and clinical studies have demonstrated that OVTs have oncolytic properties and can stimulate antitumor immune responses against various malignancies (41). However, the two most challenging problems of OVTs in the process of application are as follows: (i) a significant reduction in the efficacy because of unsatisfactory oncolysis or the virions are eliminated by the body’s strong immune system and (ii) OVs may infect and damage healthy tissues and cells with the increase of viral titer (42). For these two problems, on the one hand, the curative effects can be enhanced by further modification of their genome, which named armed OVs. Mechanistically, a variety of different armed oncolytic strategies have been explored, with particular success observed in strategies introducing immune-stimulating genes (such as T-Vec has an insertion of human GM-CSF in both copies of the ICP34.5 gene within HSV-1) and tumor-damaging genes (such as the insertion of tumor-suppressor genes or RNA interference to regulate oncogenes) (43–45). Although their efficacy is more favorable, the exogenous genes introduced by armed OVs have more unforeseen effects and potentially dangerous to normal cells than original OVs. On the other hand, the selectivity of the OVs also can be enhanced by further deletion of genes which essential for their proliferation in normal cells (42). For example, the deletion of E3 gene in adenovirus or ICP47 gene in HSV-1 can promote peptide loading of MHC-I molecules to encourage the elimination of virions by the immune system in normal cells (46). However, these changes cannot affect the survival of OVs in cancer cells due to their antiviral immune responses are inherently defective. Nowadays, the third-generation adenovirus vector engineered by gene editing technology, which removes all of genes except inverted terminal repetitions (ITRs) and a packaging gene Ψ, only acts as an exogenous gene delivery system without the ability of self-replication (47, 48). Although the specificity and capacity of exogenous gene insertion are commendably increased, the absent replicative activity and low immunogenicity of virions can’t activate the immune response or very poorly so that OVs lose the abilities of oncolysis and stimulating the systemic adaptive antitumor immune response (49). Therefore, it is indispensable that these engineered OVs treat cancer patients in combination with other antitumor agents, especially ICIs and adoptive immunotherapy.

## 3 Summary of OVTs for treating GC

### 3.1 Adenovirus

#### 3.1.1 The internal structure and invasive process of adenovirus

**Figure 3:**
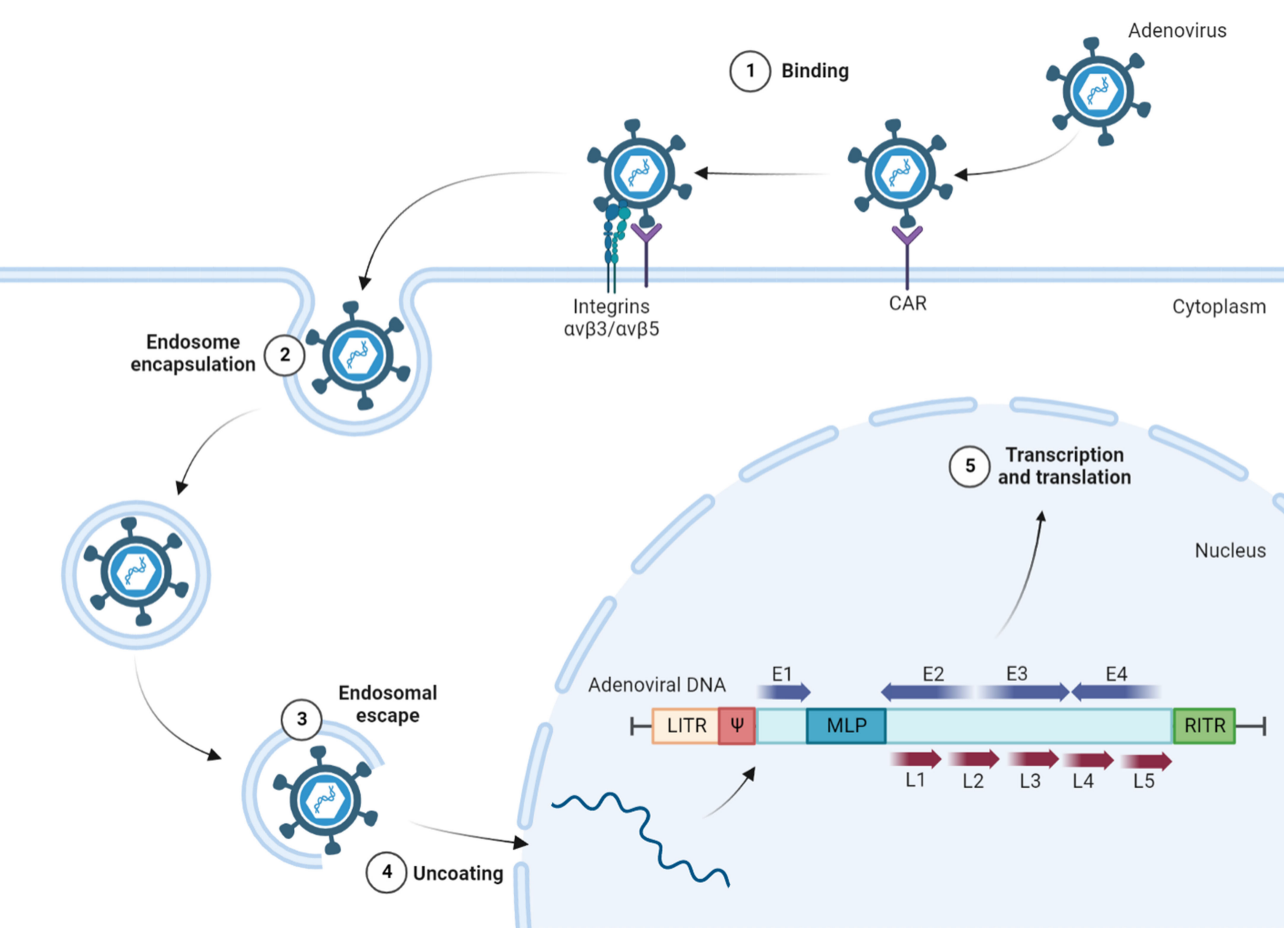
The internal structure and invasive process of adenovirus. Infection with adenovirus is mainly initiated by high affinity binding of fiber protein to CAR, with the participation of αvβ3/αvβ5 integrins as secondary receptors. Endocytic virions are released from lysosomes and transferred to the nucleus through nuclear pore complexes for transcription and translation. The adenoviral genome contains two ITRs at both ends, the packaging signal Ψ, and the major functional genes, such as early transcription units E1~E4 and late transcription units L1~L5. CAR, coxsackievirus adenovirus receptor; RGD, arginine-glycine-aspartate; LITR, left inverted terminal repetitions; RITR, right inverted terminal repetitions; Ψ, packaging signal; E1~E4, early transcription units; L1~L5, late transcription units; MLP, major late promoter.

Adenovirus is a nonenveloped double-stranded linear DNA virus with a nucleoprotein core encapsulated by an icosahedral protein capsid from which proteinaceous fibers protrude (50). It mainly initiates infection by high affinity binding of the fiber protein to CAR (**Figure 3**) or other receptors, such as CD46 and desmoglein (DSG)-2 (51). Upon cell binding, adenovirus typically requires a secondary receptor for endocytic uptake. This is usually mediated by the arginine-glycine-aspartate (RGD) sequence in an exposed loop of the penton base binding to active state αvβ3/αvβ5 integrins, followed by outside-in signals, which are critical for stimulating virion endocytosis to enter lysosomes. Because adenoviruses change the conformation of the protein capsid through a highly controlled process within lysosomes, the viral DNA are released from the lysosomes and transferred to the nucleus through nuclear pore complexes, and a series of complicated but regulated transcription and translation processes are carried out (52).

Adenoviruses contain a genome of approximately 36 kb with inverted terminal repetitions (ITRs) of ∼100 bp at both ends, and on the inside of the left ITR (LITR), the signal Ψ is involved in viral packaging. Between the right ITR (RITR) and Ψ, early transcription units E1~E4 encode proteins that are required for viral replication, and all major late proteins are organized in the transcription units L1~L5, related to the assembly of adenovirus (53). E1A is the first gene that is transcribed during adenovirus infection to regulate the metabolism of host cells to make replication easier, and E1A protein can also activate the promoters of other early transcription units (54). Replication requires a complex constructed by three viral proteins encoded by E2 genes: precursor terminal protein (pTP), DNA polymerase (DNA Pol), and the single-stranded DNA binding protein (ssDBP) (55). The E3 protein is a glycoprotein that can be transferred to the endoplasmic reticulum (ER) and then abrogate cell surface transport of MHC class I molecules to avoid the activation of CTLs (56). That, combined with the E4 gene, encodes at least 6 viral proteins that counteract host antiviral proteins during productive adenovirus infection (57). These products not only destroy the intracellular defensive capability of OVs but also block the signals of activating immune cells, ultimately promoting the proliferation of virions in host cells without restriction. When the early preparation for viral amplification is basically completed, the transcription and translation of the early transcription units are shut down, and the common major late promoter (MLP) begins to regulate the expression of late transcription units L1~L5 (58). With the participation of Ψ, mature progeny virions rush out of the tumor cells and enter the next round of lifecycle.

#### 3.1.2 As an oncolytic tool

Oncolytic adenovirus therapy is gaining importance as a novel treatment option for the management of various cancers. As a well-known OV, oncolytic adenovirus has many studies and applications in treating GC, which can replicate in and kill tumor cells selectively (**Table 1**). Multiple studies have indicated that adenovirus possesses good selectivity and infectivity in GC cells to yield oncolysis (101, 103). According to the interpretation of the invasive process, Lotta Kangasniemi et al. incorporated an RGD-containing peptide into the HI loop of the fiber knob to preferably utilize αvβ-class integrins for binding and internalization, which significantly enhanced the transduction of target cells and oncolysis (59). However, it is worth noting that the modification of ligands also can promote more virions to enter normal cells, causing unwanted off-target and side effects. In addition, adenovirus can also increase the therapeutic effectiveness of peritoneal metastasis for GC patients. Peritoneal metastasis is the most frequent form of distant metastasis and recurrence of GC, and its prognosis is extremely poor due to its resistance to systemic chemotherapy. In an orthotopic human GC peritoneal dissemination mouse model, intraperitoneal administration of adenovirus (OBP-401) enhanced the accelerated autophagy and apoptosis of malignant cells and synergistically suppressed the peritoneal metastasis of GC in combination with paclitaxel (PTX) (61).

**Table 1:** Studies of various OVs for the treatment of GC.

Role	Virus	Modification	Detail	The route of administration	Combination	Effect	Reference
As an oncolytic tool	Adenovirus	Increase infection efficiency	the fiber is modified with an integrin-targeted motif	i.p.	None	benefit gene transfer efficiency and cell killing	(59)
			the fiber is modified with an IgG Fc-binding motif from the Staphylococcus protein A	i.p.	human anti- CEA monoclonal antibody	offer a therapeutic modality against CEA-producing GC	(60)
		Engineered	OBP-401	i.p.	paclitaxel	synergistic antitumor effect	(61)
			OBP-401	no animal research	None	detect GC cells from the peritoneal washes	(62)
	HSV-1	Engineered	NV1066	i.p.	None	induce apoptosis and inhibit metastasis	(63, 64)
			G47Δ	i.t.	None	decrease M2 macrophages while increasing M1 macrophages and NK cells in TME	(65)
			G207	i.p.	None	inhibit peritoneal metastasis	(66)
			G207	i.p.	5-fluorouracil and surgical resection	synergistic antitumor effect	(67)
	VSV	Original	VSV	no animal research	None	induce apoptosis	(68)
	VACV	Engineered	GLV-1h254	no animal research	None	detect circulating tumor cells	(69)
	NDV	Engineered	NDV(F3aa)	i.p.	None	inhibit peritoneal metastasis	(70)
	Measles virus	Engineered	rMV-Hu191	i.t.	cisplatin	synergistic antitumor effect	(71, 72)
	Reovirus	Original	reovirus	i.t.	trastuzumab	augment trastuzumab-induced cytotoxicity	(73)
			reovirus	i.p.	None	inhibit peritoneal metastasis	(74)
As an exogenous gene delivery system	Adenovirus	Insert exogenous genes	WT p53	i.t.	None	selective growth inhibition in mutated p53 GC	(44)
			dominant negative IGF-Ir	i.t.	None	suppress tumorigenicity and induce apoptosis	(75)
			Bax	i.t.	None	inhibit growth and induce apoptosis	(76)
			IRF-1	no animal research	None	induce apoptosis	(77)
			Flt-1	i.p.	None	suppress peritoneal dissemination	(78)
			UPRT	i.t.	5-fluorouracil	enhance the sensitivity of 5-fluorouracil	(79)
			ICAM-2	i.t.	None	enhance the adhesion and activation of NK cells	(80)
			TRAIL	i.t.	paclitaxel	synergistic antitumor effect	(81)
			XAF1	i.t.	None	induce autophagy	(82)
			cGMP-dependent PKG II	i.p. and i.t.	None	delay growth, induce apoptosis, and inhibit metastasis and angiogenesis	(83)
			DKK1	i.t.	None	suppress tumorigenicity of cancer stem cell *via* attenuating Wnt signaling	(84)
			p33	no animal research	None	inhibit growth and induce apoptosis	(85)
			anti-p21-Ras scFv	i.v.	CIK cells	synergistically kill tumor cells	(86)
			HER2-ECD	i.p.	Trastuzumab	inhibit peritoneal metastasis	(87)
			TIPE2	i.v.	None	inhibit migration, invasion, and metastasis *via* reversal of EMT	(88)
			ING4 and PTEN	i.t.	None	synergistically suppress tumor and induce apoptosis	(89)
			NK4	i.p.	None	prevent peritoneal dissemination	(90)
			B4GALNT2	i.p.	None	prevent peritoneal dissemination	(91)
			IL-10	i.p.	None	prevent peritoneal dissemination	(92)
			HSP-gp96	no animal research	None	promote T cell and DC response	(93)
			FasL and B7-1	i.t.	None	promote the activity of CTLs	(94)
		Knock down endogenous genes	Met	i.t.	None	inhibit growth	(45)
			Mcl-1	no animal research	5-fluorouracil and cisplatin	overcome chemotherapy resistance	(95)
			PAI-1	i.p.	None	inhibit peritoneal metastasis	(96)
			PGK1	no animal research	5-fluorouracil and mitomycin	synergistically kill tumor cells	(97)
			RhoA and RhoC	i.t.	None	inhibit growth and invasion	(98)
			PI3K	no animal research	None	inhibit proliferation and enhance apoptosis	(99)
		Replace promoters	CEA promoter	no animal research	5-fluorocytosine	enhance the sensitivity of 5-fluorocytosine	(100)
			CEA promoter	no animal research	ganciclovir	confer sensitivity to ganciclovir	(101)
			hTERT promoter	i.t.	None	eliminate quiescent cancer stem-like cells	(102)
			MK promoter and Cox-2 promoter	no animal research	None	show good selectivity and infectivity in GC	(103)
			a β-catenin/T-cell factor-responsive promoter	no animal research	None	improve efficacy and reduce toxicity	(104)
		others	induce MAGE-1 into DC vaccine	i.t.	None	stimulate anti-tumor immunity specific to GC	(105)
			induce L-PGDS into MSCs	i.t.	None	MSC-derived EVs to deliver L-PGDS to treat GC	(106)
	HSV-1	Insert exogenous genes	TSP-1	i.t.	None	enhance viral oncolysis with antiangiogenesis	(107)
		Replace promoters	hTERT promoter	no animal research	None	inhibit proliferation and enhance apoptosis	(108)
	VACV	Insert exogenous genes	survivin T34A and FilC	i.p.	None	synergistic antitumor effect	(109)
			hNIS	i.t.	None	image with (99m)Tc pertechnetate scintigraphy and PET/CT	(110)
	NDV	Insert exogenous genes	rL-RVG	i.t.	None	suppress migration	(111)
			IFN-λ1	no animal research	None	change Th1/Th2 response of TME	(112)
			GFP	no animal research	None	specifically detect the spread of intraperitoneal cancer	(113)

i.p., intraperitoneal injection; i.t., intratumoral injection; i.v., intravenous injection; IGF-Ir, insulin-like growth factor I receptor; IRF-1, interferon regulatory factor-1; Flt-1, a soluble form of VEGF receptor; UPRT, uracil phosphoribosyltransferase; hTERT, human telomerase reverse transcriptase; ICAM-2, intercellular adhesion molecule-2; MAGE-1, melanoma antigen gene-1; TRAIL, TNF-related apoptosis-inducing ligand; XAF1, XIAP-associated factor 1; cGMP, cyclic guanosine monophosphate; PKG, protein kinase; DKK1, Dickkopf-1; MK, midkine; Cox-2, cyclooxygenase-2; Mcl-1, myeloid cell leukemia-1; scFv, single-chain fragment variable; CIK; cytokine-induced killer; PAI-1, plasminogen activator inhibitor-1; HER2-ECD, HER2-extracellular domain; PGK1, phosphoglycerate kinase 1; TIPE2, tumor necrosis factor-alpha-induced protein 8-like 2; EMT, epithelial-mesenchymal transition; ING4, inhibitor of growth 4; PTEN, phosphatase and tensin homolog; B4GALNT2, β1,4N-acetylgalactosaminyltransferase 2; HSP-gp96, heat shock protein-glycoprotein96; Rho, Ras homolog gene family; PI3K, phosphoinositide 3-kinase; L-PGDS, lipocalin-type prostaglandin D2 synthase; EV extracellular vesicle; TSP-1, thrombospondin-1; VSV, vesicular stomatitis virus; hNIS, human sodium iodide symporter; IFN-λ1, interferon-λ1; GFP, green fluorescent protein.

#### 3.1.3 As an exogenous gene delivery system

Adenovirus vector is also a targeted, safe, and excellent gene delivery system, enables us to introduce exogenous genes into GC at will due to its selectivity for cancer cells (**Table 1**). P53 is a suppressor of carcinogenesis that plays a crucial role in a variety of cancers, including GC (114). *In vivo* studies showed that the growth of subcutaneous tumors of p53 mutant GC cells was significantly inhibited by intratumor injection of recombinant adenovirus encoding wild-type p53 (AdCAp53), but no significant growth inhibition was detected in the growth of p53 wild type GC (44). phosphatase and tensin homolog (PTEN) tumor-suppressor activity in the PI3K/Akt/mTOR pathway is essential to regulate many cellular processes of GC, including proliferation, survival, energy metabolism, and metastasis (115). Zhang, H. et al. revealed that a recombinant adenovirus co-expressing inhibitor of growth 4 (ING4) and PTEN (AdVING4/PTEN) could synergistically induce apoptosis of GC *via* enhancement of endogenous p53 responses (89). IFN regulatory factor-1 (IRF-1), XIAP-associated factor 1 (XAF1), and cGMP-dependent protein kinase (PKG) II, as tumor suppressor genes, also can inhibit proliferation and promote apoptosis of GC in a similar way (**Table 1**). Moreover, knocking down and out oncogenes possess the similar antitumor activity in the prolongation of GC patients’ survival. The PI3K-serine/threonine kinase (AKT)-mammalian target of rapamycin (mTOR) pathway is an important cellular pathway involved in cell growth, tumorigenesis, cell invasion, and drug resistance. Bao-Song Zhu et al. constructed a recombinant adenovirus with RNA interference to silence PI3K gene. After the PI3K signaling pathway has been blocked by siRNA, the proliferation of cells was inhibited and the apoptosis of GC cells was enhanced (99). In addition, myeloid cell leukemia-1 (Mcl-1) is an antiapoptotic protein that regulates apoptosis sensitivity in many cancers. When adenovirus-mediated RNAi technology was used to knockdown the expression of Mcl-1 in GC, CD44^+^ cancer stem cell (CSC)-like cells became sensitized to chemotherapeutic agents such as 5-fluorouracil (5-FU) and cisplatin (CDDP) (95). More interestingly, a green fluorescent protein (GFP)-expressing adenovirus can detect malignant cells from the peritoneal washes of GC patients more sensitively and may thus be useful for both therapy stratification and precision medicine (62).

#### 3.1.4 Relevant clinical trials

Based on previous studies, the Sidney Kimmel Cancer Center at Thomas Jefferson University is investigating the side effects of the Ad5. F35-hGCC-PADRE vaccine and determining how well it works in treating patients with gastrointestinal adenocarcinoma in a phase IIA trial (NCT04111172). The adenovirus 5/F35-human guanylyl cyclase C-PADRE (Ad5.F35-hGCC-PADRE) vaccine may help train the patient’s own immune system to identify and kill tumor cells and prevent them from forming recurrences and metastases. In addition, a phase 1 trial that involves binary oncolytic adenovirus (CAdVEC) in combination with HER2-specific autologous CAR T-cells to treat advanced HER2-positive GC (NCT03740256) and a single arm phase 2 study of the combination of adenoviral p53 (Ad-p53) gene therapy administered intratumorally with approved ICIs in patients with recurrent or metastatic GC (NCT03544723) are underway.

### 3.2 Herpes simplex virus type 1

#### 3.2.1 The internal structure and invasive process of HSV-1

**Figure 4:**
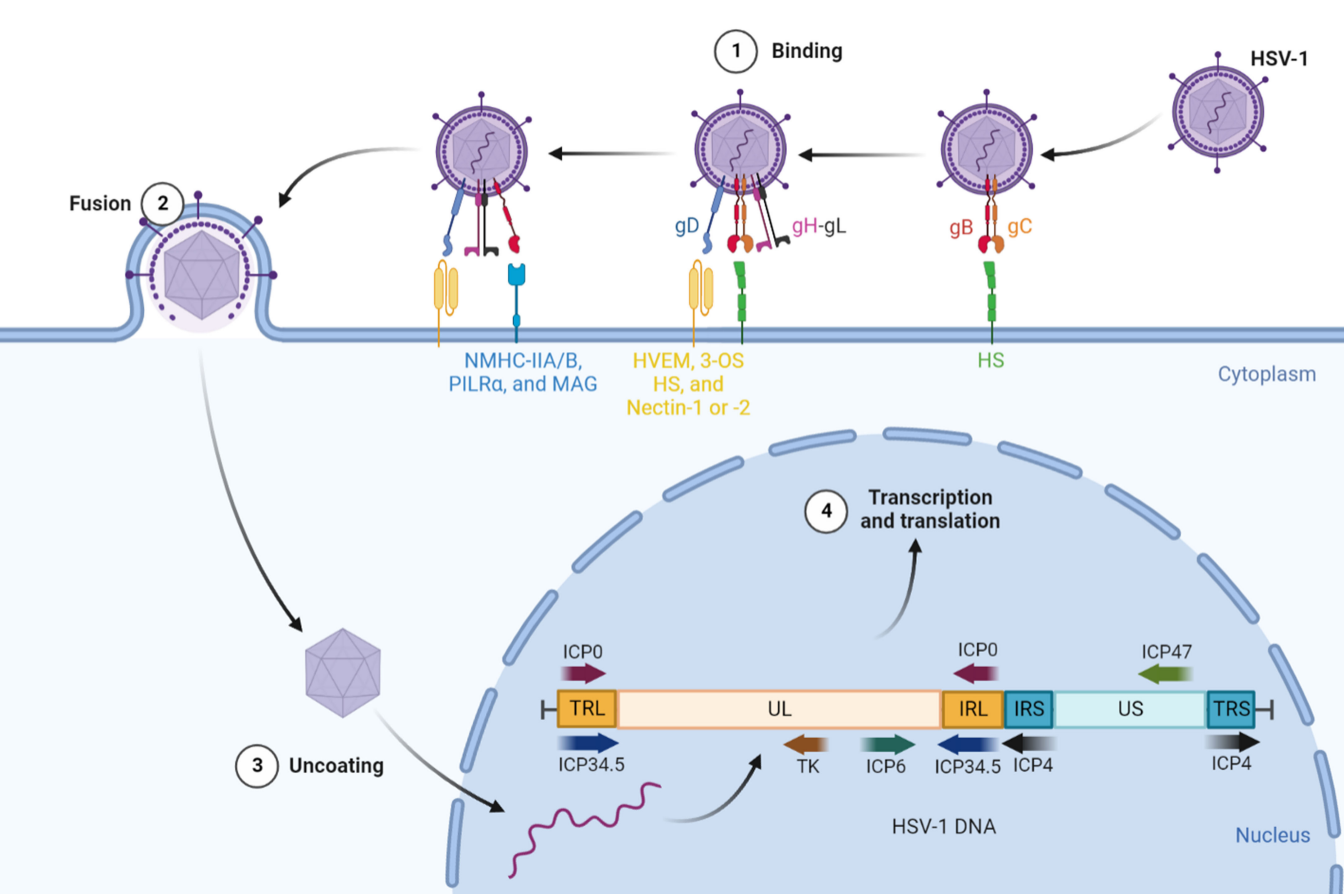
The internal structure and invasive process of HSV-1. HSV-1 requires viral binding to specific receptors to trigger membrane fusion to enter host cells. First, gB and/or gC binding to HS facilitates viral adsorption to the cells. Then, one of several entry receptors, including HVEM, Nectin-1 or -2, and 3-OS HS, can bind to gD to stabilize the attachment and promote the formation of the gH-gL complex. Subsequently, the gH–gL complex activates gB to interact with NMHC-IIA/B, PILRα or MAG. Finally, the gD, gB, and gH-gL complexes and their cognate receptors form the core fusion complex to initiate the fusion reaction of the viral envelope with the cytoplasmic membrane. The genomic DNA of HSV-1 is divided into UL, US, TRL, IRL, TRS, and IRS, which can be segmented into immediate-early (IE), early (E) and late (L) for their respective functions. HSV-1, herpes simplex virus type 1; gB, gC, gD, gH, and gL, viral entry glycoproteins; HS, heparan sulfate; HVEM, herpesvirus-entry mediator; 3-OS HS, 3-O-sulfated heparan sulfate; NMHC-IIA/B, non-muscle myosin heavy chain II A/B; PILRα, paired immunoglobulin-like receptor α; MAG, myelin-associated glycoprotein; UL/US, unique sequence of the long/short region; TRL/IRL, terminal/internal inverted repeat sequence of the long region; TRS/IRS, terminal/internal inverted repeat sequence of the short region; ICP, infected cell protein; TK, thymidine kinase.

Herpes virus is a round, enveloped double-stranded linear DNA virus with a core encapsulated by a protein capsid. The most common herpes virus, HSV-1, has a genome of 152 kb, but approximately 30 kb of these genes are not necessary for viral survival, which provides abundant space for the insertion of exogenous genes (116). Similar to most viral infection processes, HSV-1 entry into host cells requires viral binding to specific receptors to trigger membrane fusion, and multiple viral entry glycoproteins (gB, gC, gD, gH, and gL) on the surface of the virion play a coordinating role in this process (117). The direct fusion of HSV-1 with the plasma membrane of host cells involves three phases (**Figure 4**): (i) Virions attach to the membrane surface. gB and/or gC binds to heparan sulfate (HS) to facilitate viral adsorption to the cells. (ii) The host cell recognizes the virions. One of several entry receptors on the host cell surface, including HVEM, Nectin-1 or -2, and 3-O-sulfated heparan sulfate (3-OS HS), can bind to gD to stabilize the attachment between them and promote the formation of the gH-gL complex. (iii) Initiation of the fusion reaction of the viral envelope with the cytoplasmic membrane occurs. The gD, gB, and gH-gL complexes and their cognate receptors form the core fusion complex, which completes the fusion of the viral envelope with the host cell membrane (118).

Following the process of fusion, the nucleocapsid of HSV-1 is connected with the nuclear membrane to release DNA into the nucleus and activate its transcription and translation. The genomic DNA of HSV-1 is divided into long and short regions of unique sequences termed UL and US, respectively, which are flanked by regions of inverted internal and terminal repeats (119). Their expression of them is rigorous in chronological order and can be segmented into immediate-early (IE), early (E) and late (L) genes (also known as α, β, and γ). ICP0, ICP4 and ICP47 play an irreplaceable role in the early stage of infection as the expression products of IE genes. Among them, ICP0 is a multifunctional nonessential ubiquitin E3 ligase that targets a multitude of cellular proteins for proteasome-mediated degradation and counters intrinsic and IFN-related antiviral responses and epigenetic silencing of the viral genome (120). ICP4 is an essential transactivating factor that represses IE genes and activates E and L genes (120). Similar to the E3 gene in adenovirus, ICP47 can also block peptide loading of MHC-I molecules, encouraging the escape of detection by the immune system (46). The E genes include ICP6 and TK (thymidine kinase), which are mainly involved in viral DNA replication and nucleotide metabolism for amplification of HSV-1 (121). L gene expression occurs with the onset of viral DNA replication, and its products include capsid and DNA packaging proteins, glycoproteins, and tegument proteins (122). ICP34.5, one of the L genes, is the major viral neurovirulence factor, as well as a multifunctional protein that can bind phosphatase 1α (PP1α) to dephosphorylate eukaryotic translation initiation factor 2α (eIF2α) to prevent protein shutoff and bind TBK1 to block type 1 IFN induction to inhibit apoptosis of host cells (123). Thus, similar to adenovirus, we can modify the genetic composition of HSV-1 to make it more suitable for treating cancer in the clinic.

#### 3.2.2 Relevant studies and clinical trials of HSV-1

HSV-1, as an OVT, plays a pioneering role in other cancer therapies, such as T-vec for melanoma or G47Δ for glioblastoma, but its application is relatively limited in GC (**Table 1**). Firstly, HSV-1 is a wonderful oncolytic tool. The existing results suggest that simply relying on the oncolytic ability of HSV-1 can induce apoptosis of infected GC cells and effectively treat disseminated peritoneal cancers (63, 66). More meaningfully, intratumoral HSV-1 injections markedly decreased M2 macrophages while increasing M1 macrophages and natural killer (NK) cells, which means that the inherent immunosuppressive microenvironment of GC is destroyed by this method (65). Similar to adenovirus, HSV-1 can also be used to deliver exogenous genes. Thrombospondin-1 (TSP-1) suppresses tumor progression *via* multiple mechanisms, including antiangiogenesis. A novel armed oncolytic HSV-1 combined with TSP-1-mediated function, T-TSP-1, enhanced the therapeutic efficacy of GC by providing a combination of direct viral oncolysis with antiangiogenesis (107). Despite these successes in the laboratory, it is regrettable that few clinical trials using herpes virus for the treatment of GC has been conducted thus far. A phase I/II study (NCT03866525) evaluates the safety and efficacy of OH2 (an engineered recombinant herpes simplex virus) as single agent or in combination with HX008, an anti-PD-1 antibody, in patients with gastrointestinal cancers is underway.

### 3.3 Other OVs

In addition, others OVs also have corresponding therapeutic effects on GC (**Table 1**). As an oncolytic tool, Newcastle disease virus (NDV) was an effective antitumor treatment against peritoneal carcinomatosis from human GC in a xenograft model, correlated with viral replication and dosage (70, 111), and it can re-establish antitumor immunity in the suppressive TME (112). As an exogenous gene delivery system, Wang, M. et al. constructed a recombinant vaccinia virus (VACV) strain expressing mutant survivin T34A (SurT34A) and FilC and validated its strong replication and destruction ability in a murine GC model (109). In particular, due to its ability to selectively invade tumor cells, the therapeutic efficacy of a novel genetically engineered VACV carrying the human sodium iodide symporter (hNIS) gene, GLV-1 h153, was investigated in GC along with its potential utility for imaging with (99 m)Tc pertechnetate scintigraphy and ^124^I positron emission tomography (PET) (110). Furthermore, tumor cell-specific recombinant VACV can accurately detect live metastatic tumor cells in blood samples from mice bearing human tumor xenografts, as well as in blood and cerebrospinal fluid samples from patients with GC (69). These data encourage the continued investigation of OVTs for the diagnosis and staging of GC in clinical settings.

## 4 Prospects

GC, as a malignant tumor with a poor prognosis, combines a variety of adverse factors, and once it progresses to an advanced stage, almost all existing methods cannot achieve the desired therapeutic effect. Therefore, it is urgent to provide new treatment options for these patients. OVs have attracted much attention since their discovery, and their special abilities, such as selectively lysing tumor cells, remodelling the inhibitory TME, and activating the systemic antitumor immune response, have made many researchers regard OVT as a promising strategy for the treatment of cancer, and several clinical trials have commendably confirmed this hypothesis. However, to date, research on GC is still quite limited, and the few experimental studies and clinical trials cannot promote the development of OVTs for GC. Thus, for better comprehension, we combined previous results in various cancers with our own insights to discuss the prospects of OVT.

### 4.1 OV is not only a killer but also a carrier

OVs are well known for their capacity for selective oncolysis, and it is almost effortless to obtain satisfactory positive results *in vitro*. However, during the actual usage process, due to the complex internal environment in the body, the interactions among various factors results in dissatisfactory oncolysis (124). If the dose is increased, adverse phenomena such as off-target infection of normal cells and unnecessary inflammatory responses will also appear (125). After continuous modification and development, their original oncolytic ability has been increasingly marginalized, and increasing attention has been given to selective infection, as an admirable exogenous gene delivery system (126, 127). Most related studies on the introduction of exogenous genes into GC cells have been enumerated (**Table 1**). However, one important point is that GC is a molecularly and phenotypically highly heterogeneous disease (128), and the previous studies basically selected meaningful genes from other cancers and stuck them into GC, which is unfavorable for further application of the technique in the clinic. The one-size-fits-all approach is one of the key reasons for the huge differences in therapeutic efficacy among patients, and the molecular typing of various tumors can make up for this deficiency to better guide the choice of clinical medication, including for GC (129).

The Asian Cancer Research Group (ACRG) previously performed whole-genome sequencing of GC and divided it into four subtypes: microsatellite instability (MSI); microsatellite stability/epithelial-mesenchymal transition (MSS/EMT); microsatellite stability/TP53 activation (MSS/TP53^+^) and microsatellite stability/TP53 mutation (MSS/TP53^-^) (130). For example, compared with MSS/TP53^+^ GC, the MSS/TP53^-^ subtype is undoubtedly more suitable for introducing the wild-type p53 gene by OVs to remedy the error. The mutated CDH1 gene is one of the indispensable drivers of EMT involved in GC invasion and metastasis (131), and the MSS/EMT subtype has the worst prognosis because of typical CDH1 loss of expression. Thus, recovering the original level of CDH1 by OVs may improve the prognosis of GC subtype patients. Furthermore, The Cancer Genome Atlas (TCGA) project classified GC as Epstein–Barr virus (EBV)-positive (EBV), microsatellite instability (MSI), genomically stable (GS) and chromosomal instability (CIN), by analyzing gastric adenocarcinoma primary tumor tissue from 295 patients not treated with prior chemotherapy or radiotherapy (132). Taking EBV-positive GC as an example, 80% of this subtype has a PIK3CA mutation, which can cause the continuous activation of phosphatidylinositol 3-kinase (PI3K) and enhance the transmission of intracellular signals, promoting the carcinogenesis of gastric epithelial cells (133). We can reverse this process by relying on the function of OV carriers. Currently, our understanding of molecular the classification of GC has substantially changed, and the capacity of OVs to deliver exogenous genes has also been significantly enhanced. Based on both, targeted correction of characteristic abnormally expressed genes of various subtypes by OVTs can overcome the heterogeneity of GC, benefiting each patient and fulfilling the concept of precision medicine.

### 4.2 Look for more suitable promoters

**Figure 5:**
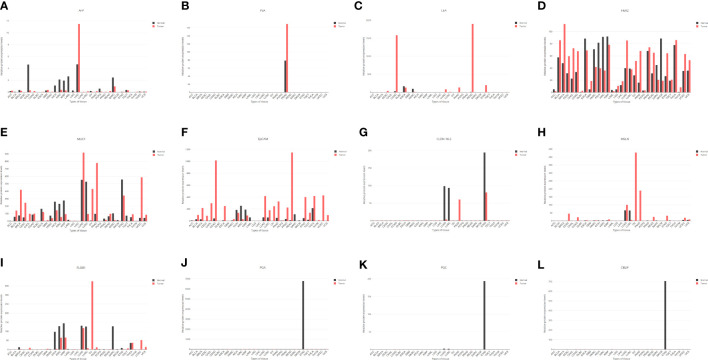
The expression levels of some genes in various tissues based on the GEPIA online database (http://gepia.cancer-pku.cn/index.html). AFP, alpha-fetoprotein; LIHC; liver hepatocellular carcinoma; PSA, prostate-specific antigen; PRAD, prostate adenocarcinoma; STAD, stomach adenocarcinoma; CEA, carcinoembryonic antigen; COAD, colon adenocarcinoma; READ, rectum adenocarcinoma; LUAD, lung adenocarcinoma; HER2, human epidermal growth factor receptor 2; MUC1, mucin 1; EpCAM, epithelial cell adhesion molecule; CLDN 18.2, claudin 18.2; MSLN, mesothelin; FOLR1, folate receptor 1; PGA, pepsinogen; PGC, gastricsin; CBLIF, cobalamin binding intrinsic factor.

The dual safety valves constituted by OVs and tumor-specific promoters can preferably avoid the occurrence of “off-target” events, further improving the targeting and safety of OVTs. For some cancers, promoters that regulate the expression of tumor-specific antigens (TSAs) are optimal candidates for OVTs; for example, the alpha-fetoprotein (AFP) promoter for hepatocellular carcinoma (**Figure 5A**) and the prostate-specific antigen (PSA) promoter for prostate cancer (**Figure 5B**) (134, 135).

Unfortunately, no appropriate promoter with impressive sensitivity and specificity has been found in GC thus far. Notwithstanding the available biomarkers of CEA, cancer antigen 19-9 (CA19-9) and cancer antigen 72-4 (CA72-4) are frequently used to monitor GC in the clinic (136). Among them, CA19-9 and CA72-4, as carbohydrate antigens, do not require specific promoters to regulate their expression, and CEA is a broad-spectrum tumor antigen mainly associated with carcinoma of the colon, lung, breast, stomach, etc (137). (**Figure 5C**). Although previous studies have shown that CEA could participate in OVTs for GC (**Table 1**), its sensitivity is not satisfactory; in fact, it is only 4.3% for early GC and 24% for AGC (138, 139). Additionally, its slight but significant upregulation in GC in the context of some inflammatory diseases suggests that the CEA promoter is not an excellent option (140). In addition, some results also suggested that HER2, mucin 1 (MUC1), epithelial cell adhesion molecule (EpCAM), claudin 18.2 (CLDN 18.2), mesothelin (MSLN), and folate receptor 1 (FOLR1) are important targets in GC (141–143). After analysis, only EpCAM seems to be a candidate tumor-specific promoter for further research, while the other promoters have either low-level expression or poor specificity (**Figures 5D-I**).

The promoters of stomach-specific proteins are capable of becoming tumor-specific promoters similar to PSA in the prostate gland, such as pepsinogen (PGA), gastricsin (PGC), and cobalamin binding intrinsic factor (CBLIF). However, during the occurrence and development of GC, a large proportion of patients will experience a process of chronic atrophic gastritis, which can decrease the expression of these proteins by damaging the gastric mucosal epithelium, causing less expression in tumors than in normal tissue (**Figures 5J-L**) (144). In brief, finding a more suitable promoter than CEA to regulate the action of OVs will be a breakthrough in the treatment of GC.

### 4.3 Overcoming the deficiency of intravenous injection

One of the vital reasons for limiting the development of OVTs is the innate antiviral immune system. Due to a general history of previous infection in the population, such as adenovirus and HSV-1, pre-existing complement, immune cells, and corresponding antibodies will rapidly neutralize and inactivate OVs in the blood circulation (145, 146). Although intratumoral injection can avoid this problem to a certain extent, in the case of systemic multiorgan metastasis, peritoneal implantation and other special circumstances, intravenous injection is still the most appropriate way to obtain a better therapeutic effect. For this reason, it is also an attractive direction to explore how to treat GC by intravenous administration without disabling the OVTs.

The complement system constitutes a complex of heat labile serum proteins and cell surface proteins that act as an innate immune defense against invading pathogens, and intravenous injection of OVs with complement regulators can counteract inactivation mediated by complement to some extent (147). For immunoglobulin, using compound targeted mutagenesis of binding sites that mediate virus-immunoglobulin interactions, the engineered OVs resisted inactivation by the aforementioned factors, avoided sequestration in liver macrophages, and failed to trigger hepatotoxicity after intravenous delivery (15).

In addition to the above two strategies, a method such as the “Trojan horse” is generally recognized as a promising solution for intravenous injection. With the help of carrier cells which have a tropism toward the TME and are susceptible to OV infection, they can remain viable long enough to allow migration and finally release OVs within the tumor bed (148). Due to their unique capability to specifically migrate to tumors, MSCs, MDSCs and tumor-infiltrating leukocytes (TILs) are universally regarded as candidates for carrying OVs, and their efficacy has been verified in glioma, colorectal cancer, and other malignancies (17, 149, 150). Moreover, angiogenesis plays an indispensable role in tumor proliferation, progression, and metastasis to supply sufficient nutrients for malignant tissues. Taking this into account, endothelial progenitor cells (EPCs) have stimulated worldwide interest as possible vehicles to perform autologous cell therapy of tumors because of their tumor-homing properties, and they may be a neotype of carriers for OVs (151).

OV delivery by carrier cells has attracted extensive attention, but there are still few studies on treating GC by this method. Only one study suggested that recombinant adenovirus KGHV500 carried by CIKs, which was equipped with a broad-spectrum anti-p21-Ras single-chain variable fragment antibody (scFv), could significantly infiltrate the TME to inhibit proliferation, migration, and invasiveness and promote cell apoptosis of GC (86). With the continuous innovation of material fabrication technology, the applications of new types of materials in cancer therapy is emerging, which can not only deliver therapeutic drugs efficiently by preventing them from being eliminated by the immune system but also target and transmit them into the tumor location relying on photodynamic therapy (PDT), magnetism, pH and so on (152–155). Therefore, using these emerging materials as carriers of OVs can be regarded as another approach to solving the problem of intravenous injection, but research on GC treatment is still lacking.

### 4.4 OVs are also diagnostic and staging tools

Diagnosing difficulty at an early stage leads to the poor prognosis of GC patients. The relevant data showed that the 5-year OS rate of patients who accepted radical surgical resection with early-stage localized GC is more than 60%, whereas that of patients with distant metastasis is less than 5% (156). In recent years, although endoscopic screening and pathological biopsy of patients with a high risk have greatly improved the diagnostic efficiency and slightly reduced the mortality of GC (157), it is undeniable that their accuracy is closely related to the technique and experience of the operators, and a number of patients are still missed due to various reasons. In addition, as an invasive operation, endoscopy also places a serious psychological burden on patients.

In summary, developing a technique with simple operation, high accuracy, and excellent patient compliance can become an important supplement to existing diagnostic methods for GC. OVs, as a tumor-targeting vector, can selectively introduce certain tagged protein genes, such as green fluorescent protein (GFP), into GC cells (62), and fluorescent endoscopy has been used in its diagnosis for a long time (158). Their combination may further improve the diagnostic sensitivity and specificity of early GC. It is widely known that therapeutic effects are closely related to the accurate staging of cancer, and patients with AGC in different stages need to receive the most appropriate therapeutic schedule to obtain the maximum benefit (159). Positron emission tomography with computed tomography (PET/CT), while currently the best method for evaluating systemic metastasis of malignant tumors, only had a sensitivity of 33% (95% CI, 17%-53%) for detecting distant metastases of GC in a multicenter prospective cohort study, which suggested it has limited additional value for GC staging (160). Based on the understanding of PET/CT imaging theory and the delivery ability of OVs, incorporating some transporter genes of radioactive substances used in PET/CT into cancer cells, such as human sodium iodide symporter (hNIS), can observably increase tracer uptake to improve the sensitivity of the examination, and this has been verified in pancreatic cancer and colon cancer (161, 162). Certainly, how to combine the advantages of OVs with existing examination methods to facilitate the diagnosis of GC is also a topic that needs additional study.

### 4.5 Destruction of the inhibitory TME by OVTs

The normal immune system possesses the function of recognizing, killing, and eliminating malignant cells in time to prevent the occurrence of cancers, which is called “immune surveillance”. However, under the selective pressure of immune surveillance, tumor cells undergo continuous remodeling at the genetic and epigenetic levels and develop a series of escape mechanisms; for example, by creating a suitable TME for growth or resisting apoptosis (163). In this long process, named “cancer immunoediting”, the immune system can both constrain and promote tumor development, which proceeds through three phases termed elimination, equilibrium, and escape to edit tumor immunogenicity and acquire immunosuppressive mechanisms (164). However, most existing immunotherapies are designed to damage the cancer cells themselves even though the dynamic and complex cell networks within the TME play a pivotal role in tumor progression and drug resistance, and it is possible to recover antitumor immunity by breaking negative and indulgent TME (165). For GC, the TME houses a variety of immunosuppressive cells, including regulatory T (Treg) cells, tumor-associated macrophages (TAMs), MSCs, MDSCs, and CAFs, which can promote tumor growth by releasing various molecules that directly activate cancer cell growth signals or reshape surrounding areas (166, 167). Accordingly, targeting these “rebellious” cells will be a new concept for treating GC, and it has been verified in many aspects.

CAFs are one of the critical components in the GC mesenchyme and not only directly confer growth advantages to cancer cells *via* paracrine signaling with chemokines, cytokines, and growth factors, but they also play a critical role in migration through direct physical interactions between CAFs and cancer cells (168). Consequently, proliferation and invasion can be significantly inhibited when the interactions between them are blocked (169). Through study of GC clinical specimens, poor prognosis and resistance to cancer therapies are closely associated with the infiltration of MDSCs, and the higher the number of MDSCs in patients with late-stage III or IV GC, the worse the prognosis (170). Inhibiting the effects of MDSCs is beneficial to GC patients (171), as well as other immunosuppressive cells (172–174). Although OVs were initially known for their ability to lyse tumor cells, they can also invade nontumor cells (150, 175). Similarly, extending their oncolytic function to tumor stromal cells can destroy the inherently inhibitory TME to restore normal immune surveillance. Applying this theory, prostate cancer and glioblastoma have achieved great curative efficacy as expected, but no similar research has been conducted on GC (176, 177).

### 4.6 Combining OVTs with other cancer therapies

To date, adjuvant therapies such as chemoradiotherapy, targeted therapy and immunotherapy alone cannot achieve revolutionary curative effects in patients with AGC, which is probably closely related to its strong heterogeneity and low immunogenicity, as previously mentioned, and a combination of various methods, including OVT, assuredly maximizes its therapeutic effects and minimizes drug resistance (178, 179). Chemotherapy as the preferred choice for postoperative and AGC patients can indeed prolong survival, and the combination of various chemotherapy regimens with OVTs shows a certain additive effect for GC as well (61, 102). Most chemotherapeutic agents were developed through their direct cytotoxic effects without consideration of their major detrimental effects on the immune system, such as lymphodepletion, an antagonism for OVTs (180). Targeted therapy has no obvious overlap with OVTs in mechanism and leads to the fact that their combination cannot complement each other (181). In addition to direct oncolysis, OVTs possess a unique ability to indirectly induce innate and adaptive antitumor immunity, which can lead to effective infiltration of immune cells, converting a “cold” tumor with few immune cells into a “hot” one with increased immune cells (182). This is extremely meaningful and not available in other immunotherapies, which means the “soldiers” against cancer cells are prepared and then need to be equipped with “weapons” to further enhance their combat capabilities at present. ICIs have revolutionized medical oncology, although currently only a subset of patients have a response to such treatment (183), and the remaining tumors are nonresponsive, in part due to a lack of tumor-infiltrating immune cells (184). Therefore, OVTs can help increase their effectiveness as a supplement to synergistically enhance the antitumor effect of ICIs, and multiple completed or ongoing items have obtained remarkable results in numerous malignancies, but not in GC (185).

Despite the robust successes of ICIs, primary and acquired resistance is common and is attributable to several factors, including insufficient antitumor T cells, inadequate function of these cells, and the impaired formation of memory T cells. CAR T-cell therapy is another form of immunotherapy that havs strong potential to address many of the limitations of ICIs by its ability to augment the number, specificity, and reactivity of T cells against tumor tissue (186). Unlike hematopoietic tumors, a key limitation of CAR T-cell therapy in solid tumors is the immunosuppressive TME, which leads to T-cell hypofunction, restricting CAR T-cells persistence within the tumors (187). Fortunately, this “door” can be opened by the “key” of OVTs. This combinatorial approach improved antitumor efficacy and prolonged survival in mouse models of solid tumors when compared with monotherapies (188). Another important hurdle encountered with CAR T-cells is tumor immune evasion due to antigen loss (187). To overcome this challenge, OVTs can restore or overexpress absent original tumor antigens and provide tumor cells with unprecedented neoantigens and any other genes that can promote the effectiveness and targeting of CAR T-cell therapy, as a target for CAR (189), as well as CAR NK-cell therapy (190). In the same way, incorporating the content of OVs to damage the suppressive TME, a combination of CAR T-cell therapy and OVTs that deliver targets of inhibitory mesenchymal cells for CAR T-cells, can disrupt the TME more favorably and completely (191). In summary, OVTs may become the optimal companion for CAR T-cell therapy to achieve unprecedented progress in treating solid tumors, including GC; nevertheless, few researchers are currently exploring this avenue.

## 5 Conclusion

OVs have attracted extensive attention and exploration worldwide because of their abilities to selectively infect and lyse tumor cells. However, in the course of decades of research, scientists found that their selectivity is not absolute, which means they can replicate and proliferate inside nontumor cells as well. Furthermore, an increasing number of animal experiments and clinical trials have revealed that not all OVs can exert ideal oncolysis under safe virus titers, limiting the development of OVTs. Nonetheless, with the continuous decryption of relevant functions of the genes in OVs, coupled with the rapid progress of gene editing technologies, artificial and purposeful modification of specific genes is possible. Currently, after varying degrees of transformation, the oncolysis of engineered OVs has been sidelined, and they are instead primarily regarded as excellent carriers of exogenous genes for targeting tumor cells. As a malignant tumor with a poor prognosis, strong heterogeneity, and low immunogenicity, how to effectively treat AGC patients has always been a worldwide problem. According to the characteristics of molecular typing in GC, engineered OVs can change their genetic and epigenetic expression levels by combining the introduction of exogenous genes and specific promoters to precisely destroy the malignancy of each subtype and preferably inform clinical medication choices. In addition, OVs can play a fascinating role in the diagnosis and staging of GC to better guide treatment in the clinic, but this needs further investigation. The TME, complicated but structured, plays an irreplaceable role in the occurrence and development of GC. By shifting the “spearhead” of OVs from tumor parenchymal cells to stromal cells, it is possible to break through this inhibitory mechanism to promote the infiltration of various immune effector cells and rebuild the function of immune surveillance against GC. For monotherapy, the crucial reasons for the poor response of various therapies are heterogeneity and resistance, so combination therapy definitely is the developmental direction of cancer treatment in the future, whether for GC or other malignancies. Specifically, the most meaningful function of OVTs is attracting immune cells into the TME to transform “cold” tumors into “hot” tumors, which will significantly improve the effect of immunotherapies for GC, such as ICIs and CAR T-cell therapy. Overall, OVTs can serve as powerful catalysts to assist other treatments, enabling GC patients to benefit more from cancer therapies.

## Author contributions

JW: writing of original manuscript. LD: revision of the manuscript. XC: language modification of the manuscript. All authors contributed to the article and approved the submitted version. All authors contributed to the conception of the study and the preparation and approval of the paper.

## Funding

This work was supported by Wenzhou Science & Technology Bureau Foundation (Grant No. Y2020144 to XC) and Zhejiang Provincial Natural Science Foundation (Grant No. LY22H160012 to LD).

## Conflict of interest

The authors declare that the research was conducted in the absence of any commercial or financial relationships that could be construed as a potential conflict of interest.

## Publisher’s note

All claims expressed in this article are solely those of the authors and do not necessarily represent those of their affiliated organizations, or those of the publisher, the editors and the reviewers. Any product that may be evaluated in this article, or claim that may be made by its manufacturer, is not guaranteed or endorsed by the publisher.

## Abbreviations

MDSC, myeloid-derived suppressor cell; CAF, cancer-associated fibroblast; MSC, mesenchymal stem cell; PAMP, pathogen-associated molecular pattern; PRR, pattern-recognition receptor; TLR, Toll-like receptor; NLR, NOD-like receptor; RLR, RIG-I-like receptor; cGAS, cyclic GMP–AMP synthase; cGAMP, cyclic GMP–AMP; TBK1, TANK­binding kinase 1; IRF3, interferon regulatory factor 3; NF-κB, the nuclear factor-κB; IFN, interferon; RIG-I, retinoic acid-inducible gene I; MAVS, mitochondrial antiviral-signaling protein; ISG, interferon-stimulated gene; CAR, coxsackievirus adenovirus receptor; HVEM, herpesvirus-entry mediator; ICD, immunogenic cell death; TAA, tumor-associated antigen; DAMP, damage-associated molecular pattern; APC, antigen-presenting cell; RGD, arginine-glycine-aspartate; ITR, inverted terminal repetition; MHC, major histocompatibility complex; PTEN, phosphatase and tensin homolog; RNAi, RNA interference; CEA, carcinoembryonic antigen; ICP, infected cell protein; EMT, epithelial-mesenchymal transition; TSA, tumor-specific antigen.
